# Comparative Biochemical and Aroma Profiling of Three Dried Chinese Mulberry (*Morus* spp.) Genotypes

**DOI:** 10.3390/ijms27104534

**Published:** 2026-05-18

**Authors:** Junrong Huang, Mesut Ada, Doğan Ergün, Müjgan Güney, Salih Kafkas, Nesibe Ebru Kafkas, Wen Yang

**Affiliations:** 1 Sericulture and Apiculture Research Institute, Yunnan Academy of Agricultural Sciences (YAAS), Kunming 661101, China; huangjunrong1981@163.com; 2 Department of Horticulture, Faculty of Agriculture, University of Çukurova, Adana 01330, Türkiye; mesutada88@gmail.com (M.A.); doganergun3@gmail.com (D.E.); salihkafkas@gmail.com (S.K.); 3 Department of Horticulture, Faculty of Agriculture, University of Yozgat Bozok, Yozgat 66100, Türkiye; mujgan.guney@yobu.edu.tr

**Keywords:** mulberry, genotype variation, dried fruit, bioactive compounds

## Abstract

This study aimed to evaluate genotype-dependent variation in biochemical composition, antioxidant capacity, and aroma profiles of dried Chinese mulberry (*Morus* spp.) genotypes. Three cultivars, Lvmeiren (green), Zhenzhubai (white), and Yunsang No 2 (red), were analyzed. Organic acids and sugars were determined using HPLC, while total phenolic content, antioxidant capacity (DPPH and FRAP), and total anthocyanins were quantified using spectrophotometric methods. Volatile compounds were analyzed by HS-SPME/GC–MS. Significant differences were observed among genotypes for all measured parameters. Among the studied genotypes, Yunsang No 2 exhibited the highest total phenolic content (379.59 mg GAE g^−1^ DW), FRAP value (21.51 μmol g^−1^ DW), and anthocyanin content (37.1 mg L^−1^). In contrast, Lvmeiren was characterized by markedly higher sucrose (22.57%) and succinic acid (3.69%) contents. Zhenzhubai exhibited the highest glucose (25.82%) and fructose (32.65%) contents, together with elevated citric (2.58%) and malic acid (2.93%) levels. Yunsang No 2 showed markedly higher total phenolics, anthocyanins, and antioxidant capacity, indicating superior nutraceutical potential. Volatile compound analysis revealed aldehydes and alcohols as dominant groups in Lvmeiren and Zhenzhubai, while acids were predominant in Yunsang No 2. Multivariate analyses (PCA and hierarchical clustering) clearly separated genotypes based on biochemical and antioxidant traits. These findings demonstrate that genotype plays a critical role in determining the nutritional quality and aroma profile of dried mulberries and provide valuable insights for breeding, cultivar selection, and functional food applications.

## 1. Introduction

Mulberry (*Morus* spp.) is an important fruit tree that has been widely cultivated in Asia for centuries. In recent years, mulberry fruits have gained attention because of their rich flavor and high nutritional value [1]. Chinese mulberries are especially valued for fresh consumption and for dried products. Drying increases shelf life and concentrates sugars, organic acids, and bioactive compounds, which makes dried mulberries popular as functional foods [2].

Mulberry fruits contain natural sugars such as glucose and fructose, organic acids including malic and citric acids, and many volatile aroma compounds that shape their characteristic taste and smell [3,4]. The balance between sugar and organic acids is very important because it determines sweetness, sourness, and overall flavor quality. Aroma compounds such as aldehydes, alcohols, esters, and terpenes also play a key role in consumer acceptance [5]. These chemical components are strongly influenced by genotype, growing conditions, and processing methods [1,4].

Mulberries are also rich in bioactive compounds, particularly anthocyanins, flavonols, phenolic acids, and other antioxidants [2,6]. Anthocyanins, which have been proven to possess strong antioxidant and anti-inflammatory properties, are responsible for the red and dark purple color in some mulberry species [7,8]. White mulberry genotypes generally contain lower anthocyanin levels, but may still have significant amounts of other phenolics and bioactive substances [4]. These compounds contribute to health benefits such as reduced oxidative stress and improved metabolic function [2,9].

Genotype is one of the most important factors affecting fruit quality traits. Genetic diversity studies using molecular markers have demonstrated substantial variability among fruit tree cultivars, which can strongly influence biochemical composition and quality characteristics [10,11]. Different mulberry cultivars show clear differences in fruit size, color, sugar composition, acidity, phenolic content, and antioxidant activity [1]. For example, red or dark-colored mulberries usually have higher anthocyanin content compared with white types [7,8]. White genotypes, on the other hand, may have higher sugar-to-acid ratios and milder flavor profiles [4]. These differences are important for both fresh consumption and processing into dried products.

Drying can significantly change the chemical profile of mulberry fruits. Water loss increases the concentration of sugars and organic acids, but heat and oxidation during drying may also reduce some volatile aroma compounds and sensitive bioactives such as anthocyanins [3,9]. Therefore, evaluating genotype-dependent variation after drying is essential to understand which cultivars maintain better flavor and functional quality.

Several mulberry genotypes, such as Lvmeiren (green fruits), Zhenzhubai (white fruits), and Yunsang No 2 (red fruits), are widely cultivated for fruit production, particularly for processing into dried fruits. Genotypes with green fruit color are unique to Chinese germplasm collections, and no reports of such genotypes have been found in the international literature. Limited information is available on how these specific genotypes differ in terms of aroma profiles, sugar composition, organic acids, and bioactive compounds after drying. Understanding these differences can help breeders select superior cultivars and assist processors in choosing suitable raw materials for high-quality dried mulberry products.

Therefore, this study aimed to investigate genotype-dependent variation in aroma, sugar, organic acid, and bioactive profiles of dried Chinese mulberries, focusing on Green, White, and Red genotypes (Lvmeiren, Zhenzhubai, and Yunsang No 2). This research will provide useful information for cultivar improvement, quality control, and functional food development.

## 2. Results and Discussion

Mulberry fruits are nutrient-rich berries containing many bioactive compounds such as sugars, organic acids, phenolics, flavonoids, amino acids, and volatile compounds that influence both nutritional value and flavor. Studies on different mulberry genotypes show large variations in these compounds, mainly due to genetic differences that affect fruit biochemical composition and functional properties [1,3,12,13,14].

### 2.1. Organic Acid Composition

Organic acid contents differed significantly among the three dried mulberry genotypes (*p* < 0.01) (Table 1). Zhenzhubai showed the highest citric acid (2.58%) and malic acid (2.93%) levels, whereas Yunsang No 2 had the lowest concentrations of citric acid and malic acid (0.53% and 2.21%, respectively). Lvmeiren exhibited intermediate citric acid content (1.17%) and a relatively high malic acid level (2.71%). Ascorbic acid was highest in Lvmeiren (0.08%), followed by Zhenzhubai (0.04%) and Yunsang No 2 (0.03%), with significant differences among genotypes. Although malic acid was the dominant organic acid in Zhenzhubai and Yunsang No 2, succinic acid was markedly higher in Lvmeiren (3.69%) compared with Zhenzhubai (1.82%) and Yunsang No 2 (1.15%).

Previous studies have shown that malic and citric acids are the predominant organic acids in mulberry fruits, which is consistent with the data obtained in this study, although this pattern was observed only in the Zhenzhubai and Yunsang No 2 genotypes [4,15,16]. Reported concentrations indicate that malic acid ranges from 1.13 to 3.04 g 100 g^−1^, citric acid from 0.48 to 1.03 g 100 g^−1^, tartaric acid from 0.15 to 0.43 g 100 g^−1^, succinic acid from 0.12 to 0.44 g 100 g^−1^, and fumaric acid from 0.01 to 0.12 g 100 g^−1^ in mulberry fruits [17,18,19]. Previous reports have also indicated that malic and citric acids significantly influence fruit acidity and flavor balance in mulberries [1,4].

In the present study, Lvmeiren exhibited a notably higher succinic acid concentration (3.69%) compared with the other genotypes. Organic acids in fruits are mainly derived from mitochondrial metabolism and the tricarboxylic acid (TCA) cycle, where succinate functions as an important intermediate metabolite. During fruit development, changes in respiratory metabolism and enzyme activity can alter the metabolic flux of the TCA cycle, leading to the accumulation of certain organic acids in specific genotypes. The elevated succinic acid concentration observed in the green-fruited genotype may therefore be associated with differences in chlorophyll-related metabolism and respiratory processes during fruit maturation. Although malic acid is generally considered the dominant organic acid in mulberry fruits, succinic acid may also occur at considerable levels and vary depending on genotype and environmental conditions [8,20,21]. Although succinic acid typically occurs at lower concentrations than malic acid, it has been reported as a minor but important contributor to flavor complexity in mulberry fruits [12]. These findings confirm that organic acid composition is strongly genotype-dependent and plays a key role in determining the sensory attributes of mulberry fruits.

### 2.2. Sugar Composition

Significant differences were observed among the genotypes for sucrose, glucose, and fructose contents (*p* < 0.01) (Table 2). Lvmeiren had the highest sucrose content (22.57%), whereas Zhenzhubai (1.97%) and Yunsang No 2 (0.53%) showed markedly lower values. In contrast, glucose and fructose were highest in Zhenzhubai (25.82% and 32.65%, respectively), followed by Lvmeiren (13.21 and 17.70%), while Yunsang No 2 exhibited the lowest concentrations (7.55% and 9.18%).

The composition of soluble sugars differed markedly among the mulberry genotypes, indicating genotype-dependent variation in carbohydrate metabolism. In the present study, fructose was the predominant sugar in Zhenzhubai and Yunsang No 2, followed by glucose, whereas sucrose was detected at relatively low levels. This pattern suggests active sucrose hydrolysis into reducing sugars during fruit maturation in these cultivars. Similar trends have been reported in mulberry fruits, where fructose commonly represents the largest proportion of soluble sugars at full maturity [14,22]. Saensouk et al. [23] also showed that fructose increases during ripening, while sucrose tends to decline as it is converted into glucose and fructose.

In contrast, the green-fruited genotype Lvmeiren exhibited a distinctly higher sucrose content than the other cultivars. Notably, Lou et al. [14] demonstrated that sugar composition in mulberries can vary substantially among cultivars, with some fully ripe cultivars such as BZM and BLM showing a sucrose-dominant profile, where sucrose accounted for up to 54% of total soluble sugars. These findings suggest that differences in sucrose synthesis, transport, and hydrolysis—regulated by enzymes such as invertases and sucrose synthase—may lead to cultivar-specific sugar accumulation patterns. Overall, the observed differences highlight the strong influence of genotype on sugar composition and sweetness characteristics in mulberry fruits.

### 2.3. Total Phenolics, Antioxidant Capacity, and Total Anthocyanins

Significant genotype-dependent differences were detected for total phenolic content, antioxidant capacity parameters, and total anthocyanin levels (*p* < 0.01) (Table 3). Yunsang No 2 exhibited the highest total phenolic content (379.59 mg GAE g^−1^ DW), markedly exceeding Lvmeiren (121.95) and Zhenzhubai (121.43).

Similarly, DPPH inhibition (%) and DPPH radical scavenging activity (%) were highest in Yunsang No 2 (62.56 and 53.89, respectively), followed by Zhenzhubai (37.48 and 28.81), while Lvmeiren showed the lowest values (24.45 and 15.77). FRAP values also differed significantly among genotypes, with Yunsang No 2 (21.51 μmol g^−1^ DW) showing substantially higher reducing power compared with Lvmeiren (4.85) and Zhenzhubai (4.29).

Total anthocyanin content was significantly greater in Yunsang No 2 (37.1 mg L^−1^) than in Lvmeiren (1.31) and Zhenzhubai (2.87). Overall, the results demonstrate pronounced genotype-related variation in phenolic composition and antioxidant capacity of dried mulberries.

Previous studies have also reported wide variability in mulberry antioxidant compounds depending on genotype. For instance, total phenolic contents in mulberries have been reported to range from 0.51–1.58 mg g^−1^ FW [24], 221 mg GAE 100 g^−1^ FW [25], and 1943–2237 mg GAE 100 g^−1^ FW in black mulberry genotypes [1]. Similarly, antioxidant activity values reported for mulberries range from 2.79–5.70 µmol TE g^−1^ FRAP [24] to 63–76% DPPH inhibition [1], while broader germplasm studies have shown antioxidant capacities of 275–1575 mg AAE 100 g^−1^ FW [26]. Anthocyanin levels also vary considerably among cultivars, with concentrations up to 8.65 mg g^−1^ DW reported for cyanidin-3-O-glucoside in mulberry fruits [13]. Such variation is largely attributed to genotype, fruit color, and metabolic differences in phenolic synthesis [27,28]. Overall, the relatively high phenolic and antioxidant values observed in Yunsang No 2 indicate that this genotype possesses strong nutraceutical potential compared with many previously reported mulberry cultivars.

### 2.4. Volatile Compound Profile

A total of aldehydes, alcohols, esters, ketones, acids, terpenes, and other minor compounds were identified in the three dried mulberry genotypes (Table 4). The relative distribution of volatile groups differed among genotypes. Aldehydes were the dominant group in Lvmeiren (43.97%) and Zhenzhubai (38.66%), whereas Yunsang No 2 showed a lower total aldehyde proportion (19.24%). Hexanal and 2-hexenal were major aldehydes in Lvmeiren and Zhenzhubai, while 3-methylbutanal was detected only in Yunsang No 2. Alcohols were highest in Zhenzhubai (40.49%), followed by Lvmeiren (28.91%) and Yunsang No 2 (8.84%). Benzyl alcohol and linalool were among the predominant alcohols, particularly in Zhenzhubai. Esters were detected at low levels overall, with the highest total ester content observed in Yunsang No 2 (3.93%), whereas Lvmeiren showed no detectable esters. Ketones were mainly represented by acetoin in Yunsang No 2 (7.67%), while Lvmeiren contained a low level of 2,3-butanedione (1.23%). Acids constituted the predominant volatile group in Yunsang No 2 (53.80%), markedly higher than in Lvmeiren (19.03%) and Zhenzhubai (12.03%). Butanoic acid and hexanoic acid were major components in Yunsang No 2. Terpenes were present at relatively low levels in all genotypes, ranging from 0.80% to 1.53%.

**Figure 1:**
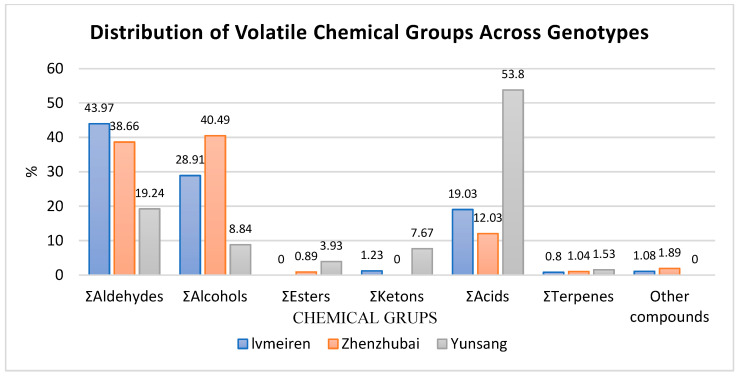
Distribution of Volatile Chemical Groups Across Genotypes.

The distribution of volatile chemical groups varied among the three dried mulberry genotypes (Figure 1). Aldehydes were predominant in Lvmeiren (43.97%) and Zhenzhubai (38.66%), whereas Yunsang No 2 exhibited a lower proportion (19.24%). Alcohols were most abundant in Zhenzhubai (40.49%), followed by Lvmeiren (28.91%) and Yunsang No 2 (8.84%).

Esters were detected at low levels across genotypes, with the highest value observed in Yunsang No 2 (3.93%). Ketones were mainly present in Yunsang No 2 (7.67%), while Lvmeiren contained a minor proportion (1.23%) and none were detected in Zhenzhubai.

Acids constituted the dominant volatile group in Yunsang No 2 (53.80%), compared with Lvmeiren (19.03%) and Zhenzhubai (12.03%). Terpenes and other compounds were detected at relatively low levels in all genotypes, with total terpene content ranging from 0.80% to 1.53%.

The volatile compound analysis further revealed clear genotype-dependent differences in aroma composition. Aldehydes were the dominant volatile group in Lvmeiren (43.97%) and Zhenzhubai (38.66%), whereas Yunsang No 2 showed a considerably lower proportion (19.24%). Compounds such as hexanal and 2-hexenal were among the major aldehydes detected in these genotypes and are known contributors to fresh green aromas in fruits. Alcohols were most abundant in Zhenzhubai (40.49%), compared with Lvmeiren (28.91%) and Yunsang No 2 (8.84%). In contrast, volatile acids dominated the aroma profile of Yunsang No 2, accounting for 53.80% of total volatiles. Previous studies have similarly reported that aldehydes, alcohols, esters, and acids represent the major volatile groups responsible for mulberry aroma characteristics [13]. Comprehensive volatile analyses have identified more than 100 aroma compounds in mulberry fruits, including esters, aldehydes, alcohols, and organic acids, highlighting the complex aromatic profile of different cultivars [14]. These results indicate that genotype plays a crucial role not only in determining biochemical composition but also in shaping the aroma profile of dried mulberry fruits.

The volatile composition observed in the present study shows both similarities and differences compared with previous reports on mulberry aroma. Zhang et al. [29] reported that aldehydes such as benzaldehyde, nonanal, and furfural were dominant volatile compounds in dried mulberries, particularly in hot-air drying (HAD) samples, whereas alcohols and ketones were more prominent in vacuum freeze-dried (VFD) products. Similarly, aldehydes were also important volatile groups in the present study, especially in Lvmeiren (43.97%) and Zhenzhubai (38.66%), confirming their major contribution to mulberry aroma. However, while [29] identified benzaldehyde (143.90 µg/kg) and nonanal (49.59 µg/kg) as dominant aldehydes, the present results highlight hexanal, 2-hexenal, and nonanal as the main aldehydes depending on genotype. Another difference concerns the volatile acid fraction. In the drying-method study, acids such as acetic acid (90–111 µg/kg) and caproic acid (46–137 µg/kg) were present but did not dominate the volatile profile, whereas in the present study acids represented the major volatile group in Yunsang No 2 (53.80%), suggesting that genotype may have a stronger influence on acid accumulation than drying conditions [30].

Odor activity value (OAV) studies further support the importance of aldehydes in mulberry aroma. Chen et al. [30] reported that compounds such as hexanal, (Z)-3-hexenal, (E)-2-hexenal, and nonanal showed very high OAVs, with hexanal and (Z)-3-hexenal reaching values up to 158.5 and 763.6, indicating their strong contribution to green and fresh sensory notes. Consistent with these findings, the present study also detected hexanal, 2-hexenal, and nonanal as major aldehydes in Lvmeiren and Zhenzhubai. In addition, compounds such as linalool and phenylacetaldehyde, which were reported by [31] as important odor-active compounds with floral and fruity notes, were also detected in the present analysis, particularly in Zhenzhubai. However, the relatively high proportion of volatile acids in Yunsang No 2 suggests a distinct aroma profile that may contribute fatty or fermented notes, indicating that both genotype and processing conditions influence the final volatile composition of mulberry fruits [29,30].

### 2.5. Multivariate Analysis of Quality Parameters

**Figure 2:**
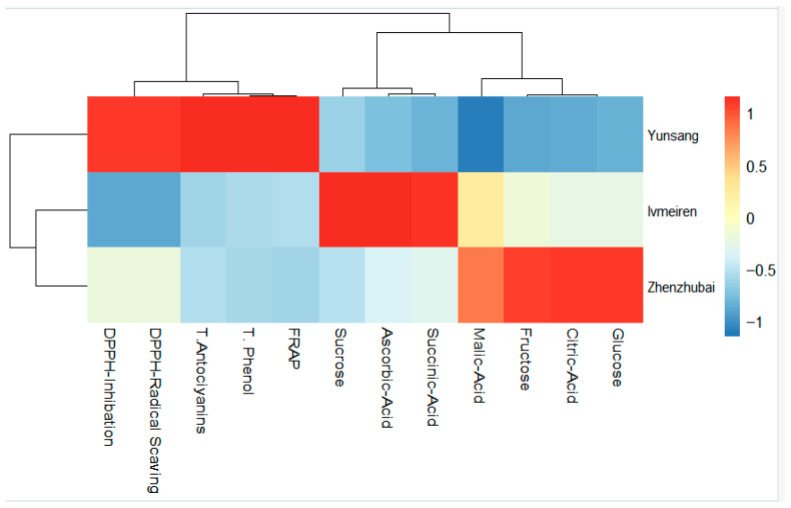
Heatmap with hierarchical clustering showing relative levels of biochemical and antioxidant traits among mulberry genotypes (Lvmeiren, Yunsang No 2, Zhenzhubai). Colors indicate standardized values (red = higher, blue = lower).

Hierarchical clustering and heatmap analysis revealed clear genotype-dependent differentiation based on biochemical and compositional traits (Figure 2). Yunsang No 2 clustered distinctly from the other genotypes and was characterized by higher relative values for total phenolics, anthocyanins, DPPH inhibition, and radical scavenging activity.

Lvmeiren formed a separate cluster associated with higher sucrose and succinic acid levels. Zhenzhubai was differentiated by relatively higher glucose, fructose, and citric acid contents.

Genotype-dependent differences in organic acid composition may be associated with variations in mitochondrial metabolism and regulation of the tricarboxylic acid (TCA) cycle during fruit development. Organic acids such as malic, citric, and succinic acids are directly linked to respiratory metabolism, and cultivar-specific differences in enzyme activities involved in the TCA cycle may influence their accumulation patterns. Previous studies have also shown that sugar and organic acid metabolism are strongly affected by developmental and genetic factors in mulberry fruits [22]. The observed genotype-associated differences in sugars, phenolics, anthocyanins, and volatile compounds may be related to cultivar-specific regulation of primary and secondary metabolic pathways. Variations in sugar composition may reflect differences in sucrose metabolism, hydrolysis, and accumulation of reducing sugars during fruit development, as previously reported among mulberry cultivars [14,23]. Similarly, differences in phenolic and anthocyanin contents are likely associated with genotype-dependent regulation of the phenylpropanoid and flavonoid biosynthetic pathways, particularly in colored mulberry fruits [13,27]. Volatile compound variation may also be linked to differences in lipid-derived aldehyde and alcohol formation, amino acid metabolism, and postharvest biochemical transformations, which have been shown to influence mulberry aroma profiles [29,30]. Therefore, the biochemical and aroma differences observed in the present study may reflect integrated effects of genotype-associated metabolic regulation.

### 2.6. Principal Component Analysis (PCA)

**Figure 3:**
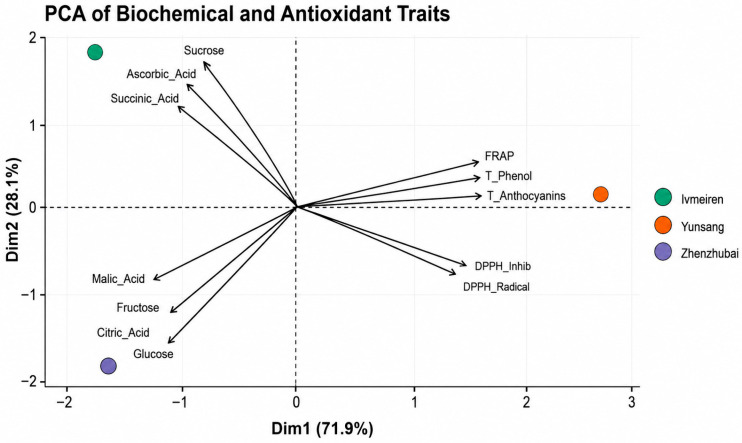
PCA biplot of biochemical and antioxidant traits in mulberry genotypes (Lvmeiren, Yunsang No 2, Zhenzhubai). PC1 and PC2 explained 71.9% and 28.1% of the total variance.

Principal component analysis revealed clear separation among the three mulberry genotypes based on biochemical and antioxidant traits (Figure 3). The first two principal components explained 100% of the total variance, with PC1 accounting for 71.9% and PC2 for 28.1%.

PC1 was mainly associated with FRAP, total phenolics, total anthocyanins, DPPH inhibition, and DPPH radical scavenging activity, which were positively correlated. Yunsang No 2 was positioned on the positive side of PC1, indicating higher relative values for these parameters.

PC2 was associated primarily with sucrose, ascorbic acid, and succinic acid, which were positively related and contributed to the separation of Lvmeiren. In contrast, glucose, fructose, citric acid, and malic acid were grouped together and contributed to the positioning of Zhenzhubai on the negative side of PC1 and PC2.

Multivariate analyses further supported the genotype-dependent differentiation observed among the three mulberry genotypes. Principal component analysis explained 100% of the total variance, with PC1 accounting for 71.9% and PC2 explaining 28.1%. PC1 was mainly associated with phenolic compounds, anthocyanins, and antioxidant parameters, positioning Yunsang No 2 on the positive side of this axis, while PC2 was associated primarily with sucrose, ascorbic acid, and succinic acid, contributing to the separation of Lvmeiren. Similar multivariate approaches have been successfully used to distinguish mulberry cultivars based on integrated biochemical and antioxidant traits [4]. Other multivariate studies have similarly shown that principal component analysis can effectively explain more than 80% of total variation among mulberry cultivars when multiple biochemical and morphological traits are considered simultaneously [31].

These findings highlight the importance of genetic variation in shaping both nutritional quality and sensory properties of mulberry fruits. Similar genetic diversity and marker-based characterization studies have also been widely used in fruit crops to support breeding and cultivar identification [10,11,32].

Overall, the present results clearly demonstrate that biochemical composition and aroma characteristics of dried mulberries are strongly influenced by genotype. Lvmeiren was characterized by extremely high sucrose and succinic acid levels, Zhenzhubai exhibited the highest glucose and fructose concentrations, whereas Yunsang No 2 showed remarkably high phenolic and anthocyanin contents together with strong antioxidant capacity. Previous evaluations of mulberry germplasm from different regions have also reported wide biochemical variation among genotypes, highlighting the importance of genetic diversity for improving fruit quality and functional value [26,33].

## 3. Materials and Methods

### 3.1. Materials

The samples belonged to three different Chinese mulberry genotypes: Lvmeiren (Green type), Zhenzhubai (White type), and Yunsang No 2 (Red type), which were obtained from the Sericulture and Apiculture Research Institute, Yunnan Academy of Agricultural Sciences (YAAS), Yunnan, China. Zhenzhubai trees were 12 years old and cultivated at a planting distance of 2.5 × 0.6 m, whereas Lvmeiren trees were 7 years old and grown at a spacing of 4 × 3 m. Thirteen years old Yusang No 2 trees were grown at 2.5 × 0.6 m. Fruits were harvested from the research orchards of the institute and dried using a hot-air drying method at 55 °C until reaching a stable moisture content before biochemical and aroma analyses.

The dried fruits were visually inspected to confirm differences in color corresponding to their genotypes (greenish, white, and red). After purchase, the samples were transported to the laboratory in sealed polyethylene bags to avoid moisture absorption. All samples were stored at room temperature (approximately 20–25 °C) in a dry and dark environment until further analysis.

Before analysis, dried fruits from each genotype were manually cleaned to remove damaged or contaminated pieces. The samples were then ground into fine powder using a laboratory grinder. The powdered samples were stored in airtight containers at −20 °C until chemical analyses of aroma compounds, sugars, organic acids, and bioactive components were performed.

### 3.2. Methods

#### 3.2.1. Determination of Organic Acid Contents

Organic acid analysis was performed according to the HPLC method developed by Bozan et al. [34]. Organic acids were extracted from ground samples using a 3% metaphosphoric acid solution. After filtration, followed by vortexing and centrifugation, the samples were prepared for injection and analysis. Organic acids were determined in triplicate using an HPLC system (Shimadzu Corporation, Kyoto, Japan, Prominence LC-20A) equipped with a UV detector and a Transgenomic (Transgenomic Inc., Omaha, NE, USA) 87H column (7.8 × 300 mm). Organic acids were then quantified using a calibration curve obtained from standard reference compounds.

#### 3.2.2. Determination of Sugar Content

Sugar analysis was performed according to the method described by Miron and Schaffer [35], with slight modifications. The extraction step was performed using ultrapure water. Then, the mixture was placed in an ultrasonic water bath for 15 min to ensure complete extraction of soluble sugars. The samples were then centrifuged, and the supernatant was collected. The clear upper phase was filtered through a membrane filter and transferred into HPLC vials for analysis. Sugar determination was carried out in triplicate using an HPLC system (Shimadzu, Prominence LC-20A) equipped with a refractive index detector (RID) and a Coregel-87C (Transgenomic Inc., Omaha, NE, USA) column (7.8 × 300 mm). The identification and quantification of sugars were performed by comparing retention times with those of external standards using a calibration curve.

#### 3.2.3. Preparation of Extracts for Bioactive Compounds Determination

The extraction of bioactive compounds from ground samples was performed using 80% methanol. The mixtures were thoroughly vortexed and incubated in an ultrasonic bath at 40 °C for 2 h, and then centrifuged at 4000 rpm for 10 min. After centrifugation, the supernatant was collected and used for further analyses.

#### 3.2.4. Total Phenolic Content

Total phenolic content was determined using the modified Folin–Ciocalteu method described by Spanos and Wrolstad [36]. Briefly, 50 µL of the extract was transferred into a 2 mL Eppendorf tube. Then, 100 µL of Folin–Ciocalteu reagent was added, followed by 1500 µL of ultrapure water. Afterward, 50 µL of 20% Na_2_CO_3_ solution was added, and the samples were incubated in the dark for 2 h. The absorbance was then measured at 760 nm using a Thermo MultiScan Go spectrophotometer (Thermo Fisher Scientific, Vantaa, Finland). Total phenolic content was calculated from a calibration curve prepared using gallic acid and expressed as gallic acid equivalents (GAE).

#### 3.2.5. Total Antioxidant Capacity (DPPH Assay)

Total antioxidant capacity was determined using the DPPH radical scavenging method described by Brand-Williams et al. [37]. Fifty microliters of extract were placed into a 2 mL Eppendorf tube, and 1950 µL of freshly prepared 0.06 mM DPPH (2,2-diphenyl-1-picrylhydrazyl) solution was added under dark conditions. In the control, 50 µL ultrapure water, instead of the sample, was added to 1950 µL DPPH solution. The blank was 80% methanol. The absorbance of samples, control and blank was then measured at 515 nm using a spectrophotometer.

DPPH inhibition (%) was calculated as:

% DPPH inhibition = [(Control absorbance − (Sample absorbance − Blank absorbance))/Control absorbance] × 100.

#### 3.2.6. Determination of Ferric-Reducing Antioxidant Power (FRAP)

Total antioxidant capacity was also determined using the Ferric-Reducing Antioxidant Power (FRAP) assay according to Benzie and Strain [38]. Fifty µL of the extracted samples was transferred into test tubes, and 2950 µL of freshly prepared FRAP reagent was added. The reaction mixture (containing Acetate buffer, 20 mM FeCl_3_ solution, 10 mM TPTZ solution) was incubated for 30 min in the dark, with tubes wrapped in aluminum foil. Absorbance was measured at 593 nm using a spectrophotometer. Results were calculated using a calibration curve prepared with Trolox standard solutions.

#### 3.2.7. Determination of Volatile Compounds

For volatile compound analysis, 1 g of ground sample was transferred to a headspace vial and mixed with 1 mL of CaCl_2_ solution. The sample was first equilibrated at 45 °C for 10 min. Following equilibration, headspace volatiles were absorbed onto a 2 cm three-phase SPME fiber coated with divinylbenzene/carboxen/polydimethylsiloxane (DVB/CAR/PDMS, 50/30 μm film thickness; Supelco, Bellefonte, PA, USA) for 50 min at 45 °C [39]. Desorption of the adsorbed volatiles was achieved by inserting the fiber into the gas chromatograph injection port maintained at 250 °C in splitless mode for 5 min. The entire sampling process was automated using a Gerstel autosampler (Gerstel Inc., Linthicum, MD, USA). Analysis of volatile flavor constituents was carried out using a Shimadzu (Shimadzu Corporation, Kyoto, Japan) GC-2010 Plus gas chromatography system coupled with a mass spectrometer (GC–MS). Separation was achieved on an HP-5 MS capillary column (Agilent, 60 m × 0.25 mm i.d., 0.25 µm film thickness), with helium employed as the carrier gas. The oven temperature was initially set to 40 °C, followed by a programmed increase to 260 °C at a rate of 5 °C/min, where it was maintained for 40 min. The injector temperature was kept at 250 °C, and the mass spectrometer was operated under electron impact ionization at 70 eV. The scanning mass range was *m*/*z* 30–400. Identification of volatile compounds was performed by comparing mass spectra with reference libraries (Wiley, NIST, and Flavor GC–MS Libraries). Further confirmation was achieved by comparison with authentic standards and published retention indices. Quantification was expressed as relative percentage values, calculated from peak areas in the total ion chromatograms (TIC).

#### 3.2.8. Statistical Analysis

All analyses were performed in triplicate, and the results are expressed as mean ± standard deviation. Data were subjected to one-way analysis of variance (ANOVA) to evaluate significant differences among mulberry genotypes. Mean comparisons were performed using the Least Significant Difference (LSD) test at significance levels of *p* ≤ 0.05 and *p* ≤ 0.01, depending on the parameter evaluated. ANOVA was conducted using JMP software 8.0.1 (SAS Institute Inc., Cary, NC, USA). Multivariate statistical analyses, including principal component analysis (PCA) and hierarchical clustering with heatmap visualization, were performed using the R software environment version 4.3.1 (R Foundation for Statistical Computing, Vienna, Austria).

## 4. Conclusions

The present study revealed unique biochemical and volatile aroma profiles in Chinese mulberry genotypes with a significant effect of genetic background. Yunsang No 2 was characterized by higher phenolic, anthocyanin, and antioxidant levels, whereas Lvmeiren and Zhenzhubai showed distinct sugar and organic acid compositions associated with flavor-related quality traits. In addition, HS-SPME/GC–MS analysis showed clear differences in volatile compound distribution among genotypes, particularly in aldehydes, alcohols, and acids, indicating substantial aroma diversity in dried mulberries. Multivariate analyses further supported the clear separation of genotypes based on integrated biochemical and antioxidant characteristics. Overall, these findings contribute to a better understanding of the metabolic and aromatic variability of mulberry germplasm and may support future cultivar selection and quality-oriented utilization of dried mulberry fruits.

## Figures and Tables

**Table 1:** The organic acid content in different studied dried mulberry genotypes (%).

Traits	Citric Acid	Malic Acid	Succinic Acid	L-Ascorbic Acid
Lvmeiren	1.17 ± 0.01 ^b^	2.71 ± 0.04 ^b^	3.69 ± 0.01 ^a^	0.08 ± 0.01 ^a^
Zhenzhubai	2.58 ± 0.01 ^a^	2.93 ± 0.13 ^a^	1.82 ± 0.05 ^b^	0.04 ± 0.01 ^b^
Yunsang No 2	0.53 ± 0.02 ^c^	2.21 ± 0.01 ^c^	1.15 ± 0.04 ^c^	0.03 ± 0.01 ^c^
LSD%1	0.03	0.152	0.074	0.01

Values represent mean ± standard deviation. Different letters within the same column indicate significant differences according to the Least Significant Difference (LSD) test at *p* ≤ 0.01.

**Table 2:** Individual sugar content in different studied dried mulberry genotypes.

Traits	Sucrose	Glucose	Fructose
Lvmeiren	22.57 ± 0.02 ^a^	13.21 ± 0.17 ^b^	17.70 ± 0.09 ^b^
Zhenzhubai	1.97 ± 0.02 ^b^	25.82 ± 0.16 ^a^	32.65 ± 0.04 ^a^
Yunsang No 2	0.53 ± 0.01 ^c^	7.55 ± 0.23 ^c^	9.18 ± 0.03 ^c^
LSD%_1_	0.037	0.379	0.115

Values represent mean ± standard deviation. Different letters within the same column indicate significant differences according to the Least Significant Difference (LSD) test at *p* ≤ 0.05.

**Table 3:** Bioactive compound content in different studied dried mulberry genotypes.

Traits	T. Phenol mg/gGAE Dw	DPPH Inh. %	FRAP μmo/gDW	T. Anthocyanins mg/L
Lvmeiren	121.95 ± 1.82 ^b^	24.45 ± 3.39 ^c^	4.85 ± 0.37 ^c^	1.31 ± 0.33 ^b^
Zhenzhubai	121.43 ± 1.78 ^b^	37.48 ± 2.15 ^b^	4.29 ± 0.02 ^b^	2.87 ± 0.38 ^b^
Yunsang No 2	379.59 ± 8.56 ^a^	62.56 ± 1.14 ^a^	21.51 ± 0.17 ^a^	37.1 ± 2.54 ^a^
LSD%1	10.304	4.816	0.473	2.99

Values represent mean ± standard deviation. Different letters within the same column indicate significant differences according to the Least Significant Difference (LSD) test at *p* ≤ 0.05.

**Table 4:** Volatile compounds identified in dried fruits of different mulberry genotypes.

R.T.	Compounds	R.I.	R.I_REF_	Lvmeiren	Zhenzhubai	Yunsang No 2
	**Aldehydes**					
6.67	3-Methylbutanal	657	646	0	0	6.06
9.87	Hexanal	795	801	14.56	19.54	4.92
11.21	2-Hexenal	850	853	10.29	10.8	0
12.38	Enanthaldehyde	898	899	1.11	1.22	1.02
16.89	Nonanal	1100	1102	7.89	4.99	5.6
15.75	Benzeneacetaldehyde	1046	1050	8.67	1.43	0
18.74	Decanal	1201	1208	1.45	0.68	1.64
	ΣAldehydes			43.97	38.66	19.24
	**Alcohols**					
8.29	Isoamyl alcohol	726	-	0	0	1.59
9.16	1-Pentanol	759	768	3.2	6.02	2.16
11.52	1-Hexanol	863	867	6.5	7.04	0
15.25	2-Ethyl-1-hexanol	1024	1026	0.83	1.25	1.57
15.47	Benzyl alcohol	1033	1035	9.29	10.94	1.92
16.81	Linalool	1074	1103	4.74	15.24	0
17.22	Benzeneethanol	1116	1116	3.57	0	1.6
22.36	β-Copaen-4-α-ol	1394	1565	0.78	0	0
	ΣAlcohols			28.91	40.49	8.84
	**Esters**					
14.6	ethyl capronate	993	-	0	0.89	3.04
18.38	Benzoic acid, ethyl ester	1174	1170	0	0	0.89
	ΣEsters			0	0.89	3.93
	**Ketones**					
5.61	2,3-Butanedione	609	-	1.23	0	0
	ΣKetons			1.23	0	0
	**Acids**					
8.65	2-methyl-Propanoic acid	742	790	0	0	0.93
9.61	Butanoic acid	784	790	0	0	19.56
10.77	Isovaleric acid	869	875	0	0	7.9
11.65	Pentanoic acid	869	915	5.85	2.35	0
13.98	Hexanoic acid	969	984	9.81	5.37	12.74
17.91	Benzoic acid	1151	1193	0	0	2.76
17.97	Octanoic acid	1154	1170	0	0.7	0
19.21	2-ethyl-Butanoic acid	1218	-	1.42	1.06	7.03
19.82	Nonanoic acid	1251	1275	1.95	2.55	2.88
	ΣAcids			19.03	12.03	53.8
	**Terpenes**					
15.32	p-cymene	1026	1025	0	1.04	0
19.37	Germacrene D	1226	1480	0	0	1.53
23.12	α-Himachalene	1310	1428	0.8	0	0
	ΣTerpenes			0.8	1.04	1.53
	**Other compounds**					
14.55	5-Isoprenyl-2-methyl-2-vinyl-tetrahydrofuran	991	-	0	1.89	0
7.73	Acetoin	702	-	0	0	7.67
23.64	γ-Dodecalactone	1472	1647	1.08	0	0

R.I.: calculated retention index; R.I_REF_: retention index obtained from the NIST library under the same chromatographic conditions.

## Data Availability

The data presented in this study are available from the corresponding authors upon reasonable request.

## Abbreviations

The following abbreviations are used in this manuscript:

ANOVA	Analysis of Variance
DPPH	2,2-Diphenyl-1-picrylhydrazyl
FRAP	Ferric-Reducing Antioxidant Power
GAE	Gallic Acid Equivalents
GC–MS	Gas Chromatography–Mass Spectrometry
HPLC	High-Performance Liquid Chromatography
HS-SPME	Headspace Solid-Phase Microextraction
SPME	Solid-Phase Micro Extraction
LSD	Least Significant Difference
PCA	Principal Component Analysis
RID	Refractive Index Detector
TCA	Tricarboxylic Acid
TIC	Total Ion Chromatogram
